# Multiplex Analysis of Serum Cytokine Profiles in Systemic Lupus Erythematosus and Multiple Sclerosis

**DOI:** 10.3390/ijms232213829

**Published:** 2022-11-10

**Authors:** Mark M. Melamud, Evgeny A. Ermakov, Anastasiia S. Boiko, Daria A. Kamaeva, Alexey E. Sizikov, Svetlana A. Ivanova, Natalia M. Baulina, Olga O. Favorova, Georgy A. Nevinsky, Valentina N. Buneva

**Affiliations:** 1 Institute of Chemical Biology and Fundamental Medicine, Siberian Branch of the Russian Academy of Sciences, 630090 Novosibirsk, Russia; 2 Mental Health Research Institute, Tomsk National Research Medical Center of the Russian Academy of Sciences, 634014 Tomsk, Russia; 3 Institute of Clinical Immunology, Siberian Branch of the Russian Academy of Sciences, 630099 Novosibirsk, Russia; 4 Pirogov Russian National Research Medical University, 117997 Moscow, Russia

**Keywords:** cytokine profile, cytokine network, chemokine, interleukin, growth factor, systemic lupus erythematosus, multiple sclerosis, correlation network, autoimmunity

## Abstract

Changes in cytokine profiles and cytokine networks are known to be a hallmark of autoimmune diseases, including systemic lupus erythematosus (SLE) and multiple sclerosis (MS). However, cytokine profiles research studies are usually based on the analysis of a small number of cytokines and give conflicting results. In this work, we analyzed cytokine profiles of 41 analytes in patients with SLE and MS compared with healthy donors using multiplex immunoassay. The SLE group included treated patients, while the MS patients were drug-free. Levels of 11 cytokines, IL-1b, IL-1RA, IL-6, IL-9, IL-10, IL-15, MCP-1/CCL2, Fractalkine/CX3CL1, MIP-1a/CCL3, MIP-1b/CCL4, and TNFa, were increased, but sCD40L, PDGF-AA, and MDC/CCL22 levels were decreased in SLE patients. Thus, changes in the cytokine profile in SLE have been associated with the dysregulation of interleukins, TNF superfamily members, and chemokines. In the case of MS, levels of 10 cytokines, sCD40L, CCL2, CCL3, CCL22, PDGF-AA, PDGF-AB/BB, EGF, IL-8, TGF-a, and VEGF, decreased significantly compared to the control group. Therefore, cytokine network dysregulation in MS is characterized by abnormal levels of growth factors and chemokines. Cross-disorder analysis of cytokine levels in MS and SLE showed significant differences between 22 cytokines. Protein interaction network analysis showed that all significantly altered cytokines in both SLE and MS are functionally interconnected. Thus, MS and SLE may be associated with impaired functional relationships in the cytokine network. A cytokine correlation networks analysis revealed changes in correlation clusters in SLE and MS. These data expand the understanding of abnormal regulatory interactions in cytokine profiles associated with autoimmune diseases.

## 1. Introduction

Systemic lupus erythematosus (SLE) and multiple sclerosis (MS) are chronic autoimmune diseases that affect many people worldwide. MS is associated with infiltration by immune cells of the central nervous system resulting in inflammation, demyelination, neuroaxonal white matter degeneration, and gliosis [1]. SLE in turn is a B cell-mediated autoimmune disease associated with the formation of autoantibodies against nuclear antigens and type III hypersensitivity, leading to systemic inflammation and tissue damage in various organs [2]. SLE and MS are among the most common autoimmune diseases. The global prevalence of SLE varies from region to region and ranges between 13 and 7713.5 per 100,000 [3]. The prevalence of MS is approximately 35.9 per 100,000 [4], but it is steadily increasing. Indeed, the prevalence of MS in the world increased by about 30% from 2013 to 2020 [2]. Identifying and remediating gaps in knowledge about the pathogenetic mechanisms of SLE and MS is critical to reducing the global burden of these autoimmune diseases.

SLE and MS are different autoimmune diseases, but they share some common features. There is evidence of a shared genetic background of these two autoimmune diseases, in particular an association with *HLA*, *IRF8*, *SH2B3*, and other immune genes [5,6]. Depression, fatigue, and cognitive impairment are common clinical symptoms of both diseases [7,8]. Additionally, both SLE and MS are characterized by chronic systemic inflammation [1,2]. Consequently, the concentration of several inflammatory mediators, including soluble adhesion molecules, in serum and cerebrospinal fluid is increased in MS and SLE [9]. In addition, confluent gene expression signatures caused by inflammation in different tissues have been found in MS and SLE [10]. Epidemiological evidence also suggests that Epstein-Barr virus (EBV) infection is one of the triggers for the development of SLE and MS [11]. It has been shown that immunization of mice with the EBV nuclear antigen 1 (EBNA1) led to the production of anti-DNA IgG antibodies, a hallmark of SLE [12]. On the other hand, high-affinity molecular mimicry between the EBNA1 and the central nervous system protein glial cell adhesion molecule (GlialCAM) was found to contribute to demyelination and the development of MS [13]. Thus, some common pathogenetic mechanisms can lead to the development of quite different autoimmune diseases, SLE or MS, so a comparative study of molecular markers can reveal the characteristic features of these autoimmune disorders. In addition, the discovery of similar molecular signatures may reveal crucial pathways that can be targeted by therapy.

Autoimmune diseases, including SLE and MS, are accompanied by cytokine dysregulation [1,2]. Cytokines are small protein molecules that regulate the homeostasis of the immune system and coordinate the immune response [14]. The levels of a broad spectrum of cytokines in one individual at a given time represent a cytokine profile. All cytokines that provide regulation of immune functions form a cytokine network. Changes in the cytokine profiles and cytokine networks are an integral part of any disease, including autoimmune diseases.

The role of cytokines in the progression of SLE and MS is significant because changes in the levels of these molecules disrupt intercellular interactions within the immune system. The levels of many pro- and anti-inflammatory cytokines significantly increase in SLE patients, including those undergoing pathogenetic therapy [15,16,17]. Cytokines such as interferon-α (IFNα), tumor necrosis factor-α (TNFα), transforming growth factor β (TGFβ), interleukin (IL)-2, IL-6, IL-8, IL-10, and IL-17 are associated with the pathogenesis of SLE [18]. For example, IFNα, TNF-α, IL-4, IL-6, and IL-10 stimulate autoreactive B cell activation, enhance the production of autoantibodies, and promote inflammation [19,20,21]. Changes in the concentration of some cytokines contribute to the dysregulation of other cytokines and exacerbate inflammation and tissue damage in SLE [18,19,21].

A shift in the cytokine profile compared to healthy donors has also been shown in MS studies [22]. Numerous cytokines including granulocyte-macrophage colony-stimulating factor (GM-CSF), IFNβ, IFNγ, TNFa, IL-2, IL-6, IL-10, IL-12, IL-17, IL-21, IL-22, IL-23, and others are actively involved in the pathogenesis of MS [22,23]. Moreover, IFNβ, IFNγ, and TNFα play a protective role in MS, so IFNβ is the first-line treatment for relapsing-remitting MS (RRMS) [22]. Immune cells penetrating the brain tissue are the main source of pro-inflammatory cytokines in MS. Chemokines of the CC, CXC, and CX3C families are involved in immune cell recruitment to the brain, immune activation, and neuroinflammation [24]. Growth factors including epidermal growth factor (EGF) and vascular endothelial growth factor (VEGF) have pleiotropic effects on immune cells and promote remyelination [25,26]. Nevertheless, our understanding of the deregulated cytokine networks in SLE and MS is still in its infancy. Research results are inconsistent, and conclusions about the change in cytokine profiles in SLE and MS are often based on the results of only a few cytokines. Therefore, further studies of changes in a broad spectrum of cytokines in these diseases are needed.

Functional and correlation interactions between cytokines in SLE or MS are studied much less frequently than changes in their absolute concentration. Typically, such studies consider the relationship between cytokines and specific clinical features [27,28,29]. Analysis of functional and correlation interactions between cytokines can provide valuable diagnostic information about abnormal regulatory interactions within cytokine profiles associated with autoimmune diseases [30]. Analysis of the relationships between cytokine levels can improve the understanding of the disruptions within cytokine networks leading to SLE and MS. Correlation matrices could be used for machine learning and building neural networks trained to make diagnostic predictions. Some work has already demonstrated the usefulness of neural networks for more accurate diagnosis of certain diseases based on various cytokine parameters [31,32,33].

In this work, we analyzed the cytokine profiles of 41 analytes including interleukins, chemokines, TNF superfamily members, and growth factors in patients with SLE or MS, and healthy donors. Patients in the SLE group received standard anti-inflammatory therapy, while MS patients were drug-free. Differences in serum cytokine concentrations both in MS or SLE patients compared with healthy donors and between MS and SLE patients have been studied. Functional and correlation interactions in cytokine networks have also been evaluated.

## 2. Results

### 2.1. Clinical Characteristics of Analyzed Groups

The following three groups were analyzed in this study: a group of healthy donors (HS group), a group of SLE patients (SLE group), and a group of individuals with MS (MS group). The clinical characteristics of these groups are presented in Table 1. The HS group included 36 individuals, the SLE group, 58, and the MS group, 56. The median age of the participants did not differ between the analyzed groups. However, the ratio of men and women in the SLE group differed from that in the other two groups (Table 1). This is explained by the higher prevalence of SLE among women [1]. The disease duration in SLE was higher than in MS. Most of the patients with SLE were in the stage of exacerbation of the disease. The median SELENA-SLEDAI index score was 6.5. The MS group included 20 patients with primary progressive MS (PPMS) and 36 people with relapsing-remitting MS (RRMS). The median EDSS index score was 2.5. Other data are shown in Table 1. It is important to note that SLE patients received therapies that affect the immune system (for details, see the Materials and Methods section and Supplementary Table S1).

### 2.2. Changes in Cytokine Concentrations in the Analyzed Groups

A list of abbreviations and description of the 41 analyzed cytokines is provided in Appendix A (Table A1). The cytokines consisted of interleukins (IL-1a, IL-1b, IL-1RA, IL-2, IL-3, IL-4, IL-5, IL-6, IL-7, IL-9, IL-10, IL-12P40, IL-12P70, IL-13, IL-15, IL-17A), interferons (IFNa2, IFNy), growth factors (EGF, FGF2, Flt-3L, G-CSF, GM-CSF, PDGF-AA, PDGF-AB/BB, TGFa, VEGF), TNF superfamily members (sCD40L, TNFα, TNFb), and chemokines (Eotaxin/CCL11, Fractalkine/CX3CL1, GRO/CXCL1, IP-10/CXCL10, MCP-1/CCL2, MCP-3/CCL7, MDC/CCL22, MIP-1a/CCL3, MIP-1b/CCL4, RANTES/CCL5, IL-8/CXCL8). Descriptive statistics (N, median, Q1, Q3, Min, Max) of cytokine concentrations for the analyzed groups are presented in Supplementary Table S2. These data can be used in future meta-analyses to combine with other data. Results of the analysis of the significance of differences in cytokine levels using the Kruskal–Wallis test with Dunn’s post hoc test for multiple comparisons are shown in Supplementary Tables S3 and S4. We applied Benjamini–Hochberg correction for multiple comparisons to remove non-significant differences. The data obtained are graphically presented as volcano plots reflecting the dependence of Log2 fold change when pairwise comparing the median values in two groups from -Log10 *p*-value.

**Figure 1:**
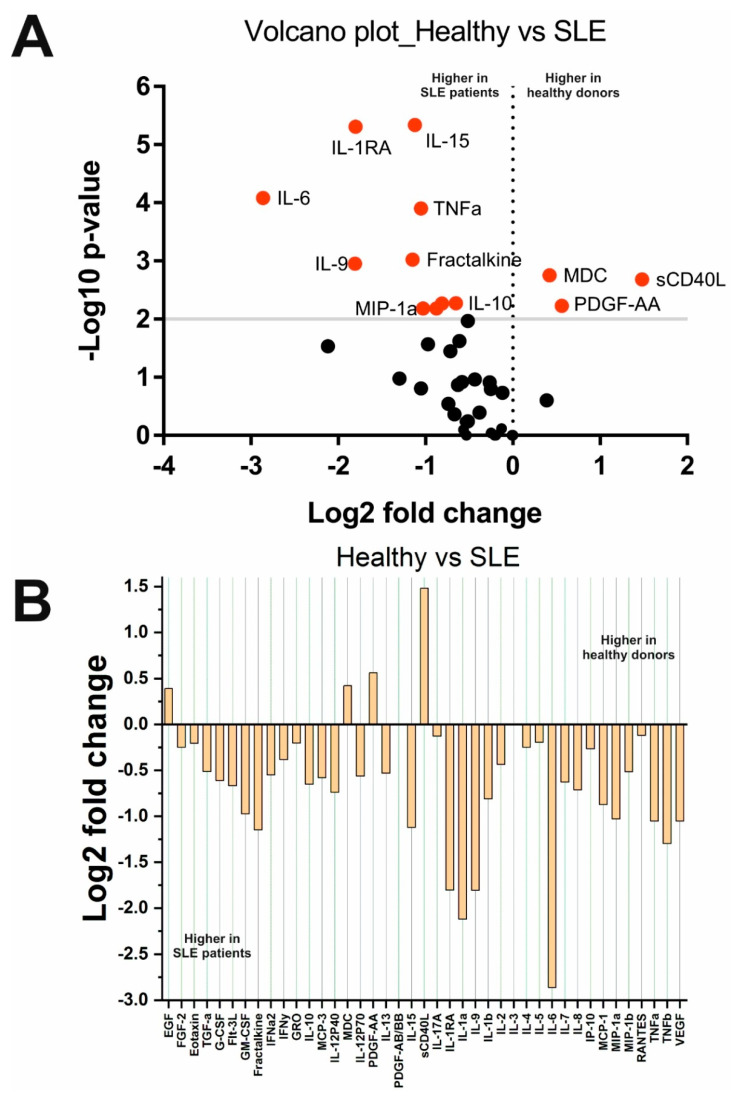
Changes in cytokine concentrations in SLE patients compared with healthy donors: (**A**) Volcano plot reflecting the dependence of Log2 fold change when pairwise comparing the median values in two groups from -Log10 *p*-value (red dots indicate significantly changed cytokines); (**B**) Log2 fold change in the concentration of 41 cytokines in the two groups.

A comparison of cytokine levels in the SLE and HS groups is shown in Figure 1. Fourteen out of forty-one cytokines changed statistically significantly. Most cytokines, IL-1b, IL-1RA, IL-6, IL-9, IL-10, IL-15, MCP-1/CCL2, Fractalkine/CX3CL1, MIP-1a/CCL3, MIP-1b/CCL4, and TNFa, were higher in SLE patients. Three cytokines, sCD40L, PDGF-AA, and MDC, were lower in SLE patients (see Figure 1). Other cytokines did not change significantly.

Analysis depending on the clinical parameters of SLE showed that the levels of IL-10 (*p* = 0.03) and MCP-1/CCL2 (*p* = 0.01) are higher in patients with a SELENA-SLEDAI index score of more than 7 than in patients with a SELENA-SLEDAI index score of less than 7. In addition, IFNy (*p* = 0.007), IL-10 (*p* = 0.02), IL-17A (*p* = 0.007), and IL-2 (*p* = 0.02) levels were higher, while IL-8 levels were lower (*p* = 0.03) in patients with a disease duration of fewer than 7 years compared with individuals suffering from SLE for more than 7 years. Therefore, the levels of IFNy, IL-10, IL-17A, and IL-2 decreased, and the level of IL-8 increased with the course of the disease. In addition, the concentration of sCD40L (*p* = 0.0009) was higher in patients with a chronic course than in patients with a subacute course. There were no changes in cytokine levels depending on sex and other clinical characteristics. This may be due to uneven sampling, particularly, as there were only 10 men and 50 women in our sample. For the same reason, it was not possible to detect differences in cytokine profiles between SLE patients with or without exacerbation of the disease (only 9 out of 60 patients were in remission).

**Figure 2:**
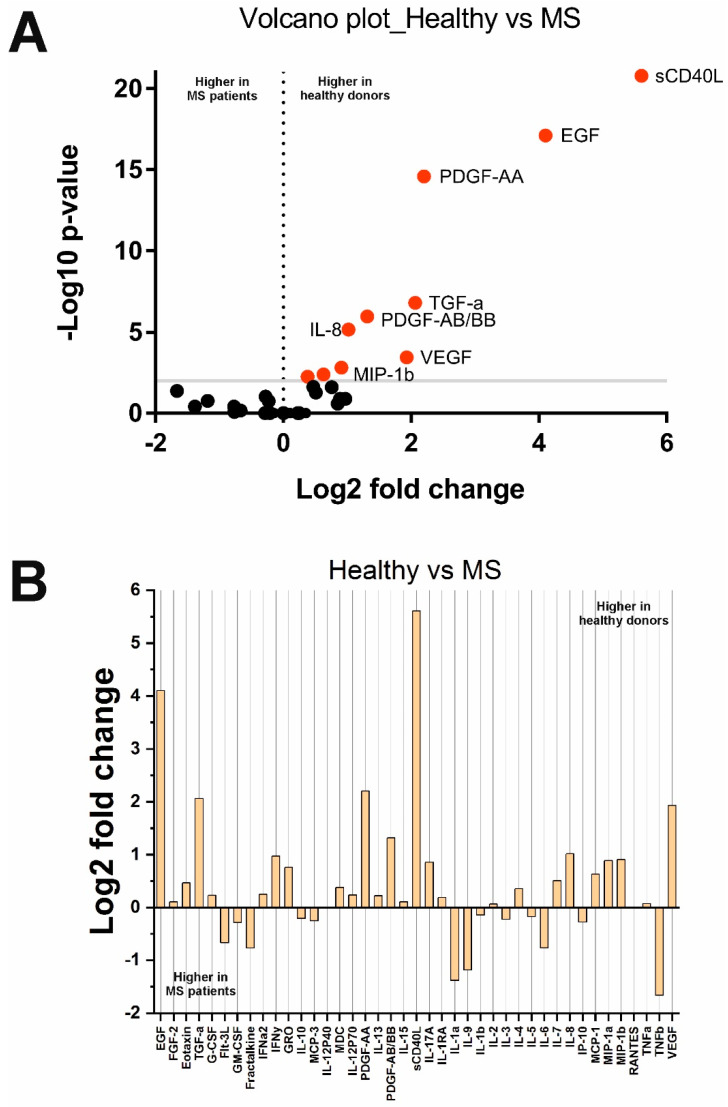
Changes in cytokine concentrations in MS patients compared with healthy donors: (**A**) Volcano plot reflecting the dependence of Log2 fold change when pairwise comparing the median values in two groups from −Log10 *p*-value (red dots indicate significantly changed cytokines); (**B**) Log2 fold change in the concentration of 41 cytokines in the two groups.

Analysis of cytokine levels in MS and HS groups showed unexpected results (Figure 2). Although some cytokines including TNFb, IL-1a, IL-9, and others, were higher in MS patients (Figure 2B), these changes were not significant. As a result, ten cytokines, sCD40L, MCP-1, MIP-1b, PDGF-AA, MDC, EGF, IL-8, TGF-a, PDGF-AB/BB, and VEGF, were found to be lower in MS patients. Interestingly, IFNα2 and PDGF-AB/BB levels were significantly higher in PPMS than in RRMS (see Supplementary Figures S1 and S2). No other significant differences were found based on the clinical data.

**Figure 3:**
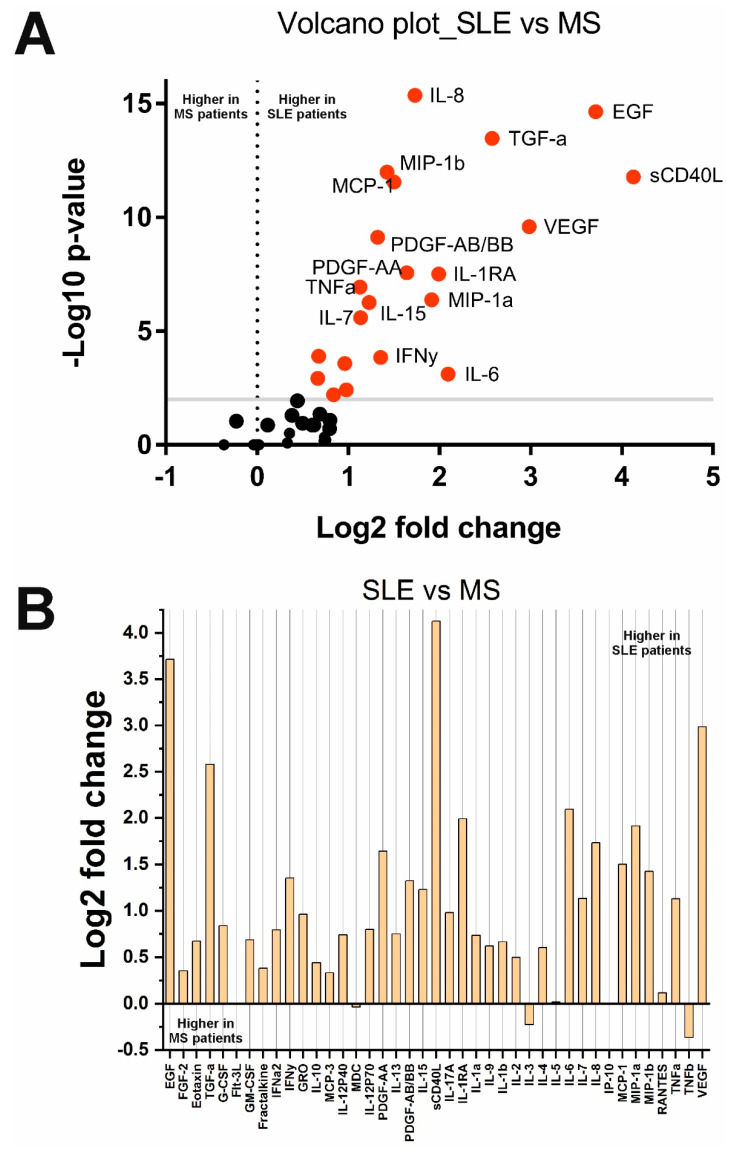
Changes in cytokine concentrations in SLE compared with MS patients: (**A**) Volcano plot reflecting the dependence of Log2 fold change when pairwise comparing the median values in two groups from -Log10 *p*-value (red dots indicate significantly changed cytokines); (**B**) Log2 fold change in the concentration of 41 cytokines in the two groups.

A comparison of cytokine levels in SLE and MS groups showed that the concentration of 22 cytokines is significantly higher in SLE patients and, accordingly, lower in MS (Figure 3). These 22 cytokines were sCD40L, MCP-1, MIP-1b, PDGF-AA, IL-1b, IL-1RA, IL-6, IL-15, EGF, MIP-1a, IL-10, TNFa, IL-8, PDGF-AB/BB, TGFa, VEGF, Eotaxin, G-CSF, GRO, IFNy, IL-7, and IL-17A. The other 19 cytokines were unchanged.

A summary list of cytokines that changed significantly (after Benjamini–Hochberg correction) in the analyzed groups is given in Table 2. A Venn diagram showing the number of overlapping significantly changed cytokines in different comparison groups is presented in Supplementary Figure S3. Interestingly, four cytokines, sCD40L, MCP-1/CCL2, MIP-1b/CCL4, and PDGF-AA, were significantly changed in all three comparisons (HS vs. SLE, HS vs. MS, and SLE vs. MS). Notably, among these cytokines, two chemokines, MCP-1/CCL2 and MIP-1b/CCL4, are altered differently in SLE and MS (Table 2). Thus, multidirectional changes in MCP-1/CCL2 and MIP-1b/CCL4 levels (increase in SLE and decrease in MS) may be the distinguishing features of these diseases. However, cytokines such as Eotaxin/CCL11, G-CSF, GRO/CXCL1, IFNy, IL-7, and IL-17A, only changed when comparing SLE vs. MS. Two cytokines (Fractalkine/CX3CL1 and IL-9) significantly changed only in SLE compared with the HS group. Alterations in these cytokines may be characteristic of SLE (increased Eotaxin/CCL11, G-CSF, GRO/CXCL1, IFNy, IL-7, and IL-17A) or MS (decreased Fractalkine/CX3CL1 and IL-9). In general, the findings indicate that SLE and MS are characterized by different changes in cytokine profiles. Cytokine dysregulation in SLE is mainly characterized by abnormalities in interleukins (IL-1b, IL-1RA, IL-6, IL-9, IL-10, and IL-15), TNF superfamily members (sCD40L and TNFa), and chemokines (MCP-1/CCL2, MIP-1b/CCL4, MIP-1b/CCL3, MDC/CCL22, and Fractalkine/CX3CL1). In MS, there is dysregulation in growth factors (PDGF-AA, TGF-a, and EGF) and chemokines (MCP-1/CCL2, MIP-1b/CCL4, MDC/CCL22, and IL-8/CXCL8).

### 2.3. Protein Interaction Network Analysis of the Significantly Altered Cytokines in MS and SLE

**Figure 4:**
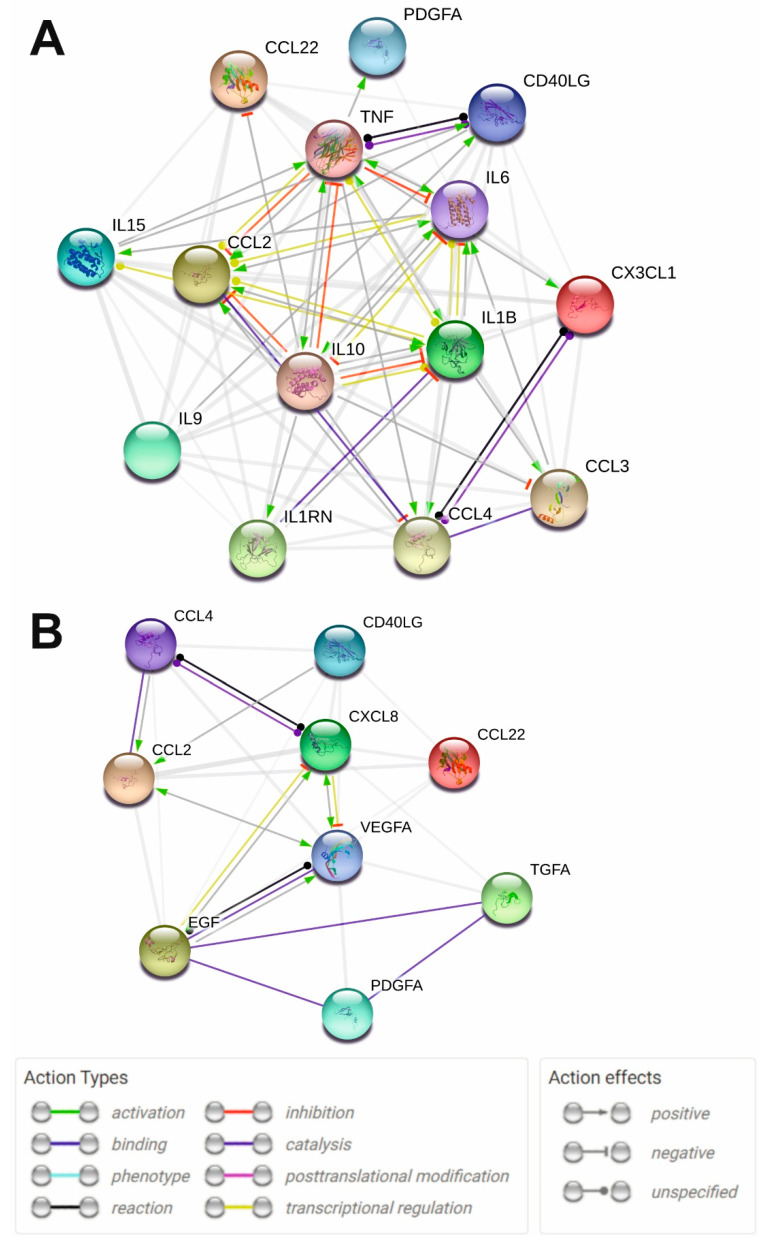
STRING protein-protein interaction networks for the significantly altered cytokines of SLE vs. HS group (**A**) and MS vs. HS group (**B**). Functional protein-protein interactions are shown. STRING 11.0 online tool (https://version-11-0.string-db.org/, accessed on 28 September 2022) was used for plotting. The legend of protein-protein interactions is shown at the bottom of the figure.

Protein interaction network analysis using the STRING 11.0 web tool allows the visualization of the functional relationships between various proteins including cytokines [34]. We constructed protein interaction networks (Figure 4) based on data on significantly altered cytokines of the SLE vs. HS group and MS vs. HS group (Table 2). The analysis showed that all significantly altered cytokines in both SLE and MS are functionally interconnected (Figure 4). Analysis of the protein interaction network in SLE showed that TNFa, IL-1b, IL-6, IL-10, and MCP-1/CCL2 have multiple functional interactions and mutually affect each other’s expression and function (Figure 4A). It can be assumed that the disruption of these cytokines is a central element in the pathogenesis of SLE. These cytokines also in turn act on chemokines including MIP-1a/CCL3, MIP-1b/CCL4, and fractalkine/CX3CL1. The core components are also functionally interconnected with other network components including sCD40L, MDC/CCL22, IL-15, PDGF-A, and IL-1RA. Thus, these data indicate that cytokine network dysregulation in SLE is primarily characterized by changes in the functional relationships of TNFa, IL-1b, IL-6, IL-10, and MCP-1/CCL2, and is generally associated with dysregulation of interleukins, TNF superfamily members, and chemokines.

In the case of MS, growth factors VEGF and EGF play an important role in pathogenesis, affecting MCP-1/CCL2 and IL-8/CXCL8 (Figure 4B). These components are connected to other network components including MIP-1b/CCL4 and sCD40L. Thus, it can be assumed that dysregulation of the cytokine network in MS is characterized by impaired functional relationships between growth factors and chemokines.

### 2.4. The Combined Multicytokine Profiles Are Different

**Figure 5:**
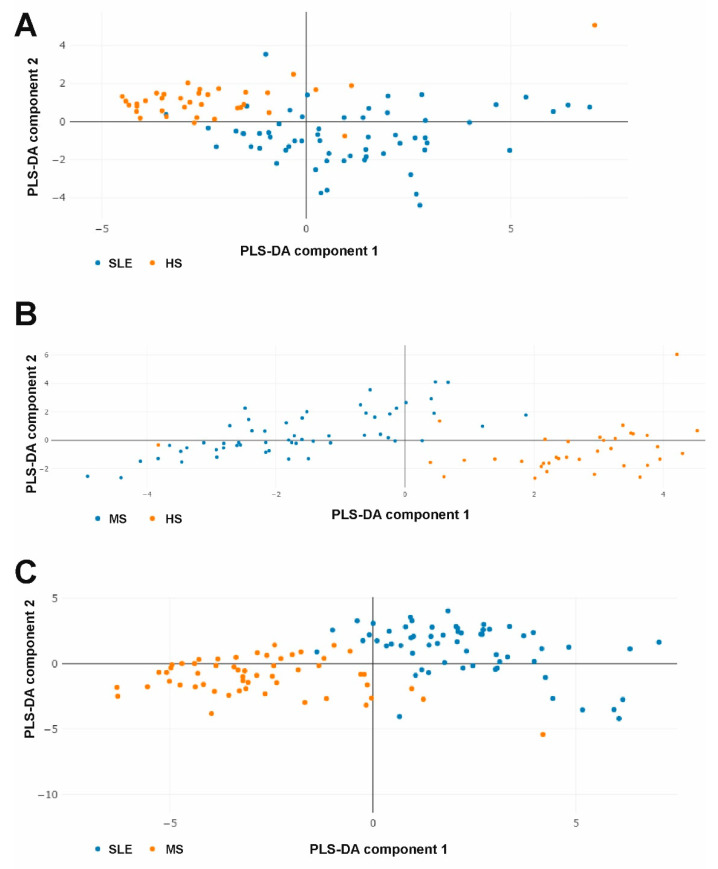
PLS-DA plots of combined multicytokine profiles when comparing SLE vs. HS (**A**), MS vs. HS (**B**), and SLE vs. MS (**C**) groups.

Using Partial Least-Squares Discriminant Analysis (PLS-DA) it is possible to pool data on all cytokines for each patient and evaluate their cytokine profiles [35]. PLS-DA results are shown in Figure 5.

The analysis showed that the data samples barely overlap in all cases (Figure 5). Thus, the PLS-DA data indicate that SLE and MS patients and healthy donors have strongly different combined multicytokine profiles. Variables importance in PLS-DA is presented in Supplementary Figures S4–S6.

### 2.5. Cytokine Correlation Networks and Correlation Clusters

**Figure 6:**
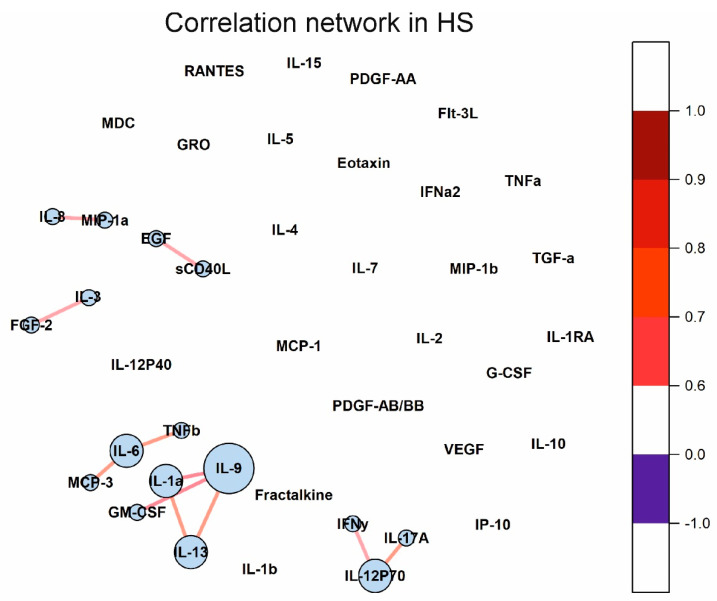
Cytokine correlation network in healthy donors. Correlations between concentrations of different cytokines are shown as colored lines. A scale showing the strength and direction of the correlation is shown in the right panel. Each cytokine is represented by a blue circle whose size is proportional to the number of statistically significant correlations.

Changes in the concentration of some cytokines can lead to changes in others. Cytokine correlation networks may reflect these changes, including those associated with cytokine dysregulation in autoimmune diseases. Correlation matrices for the three analyzed groups are given in Supplementary Tables S5–S7. The Fruchterman–Reingold algorithm was used to visualize the correlation networks [36]. The cytokine correlation network in the HS group is shown in Figure 6. It has been shown that only a few cytokines correlate with each other and form small clusters.

**Figure 7:**
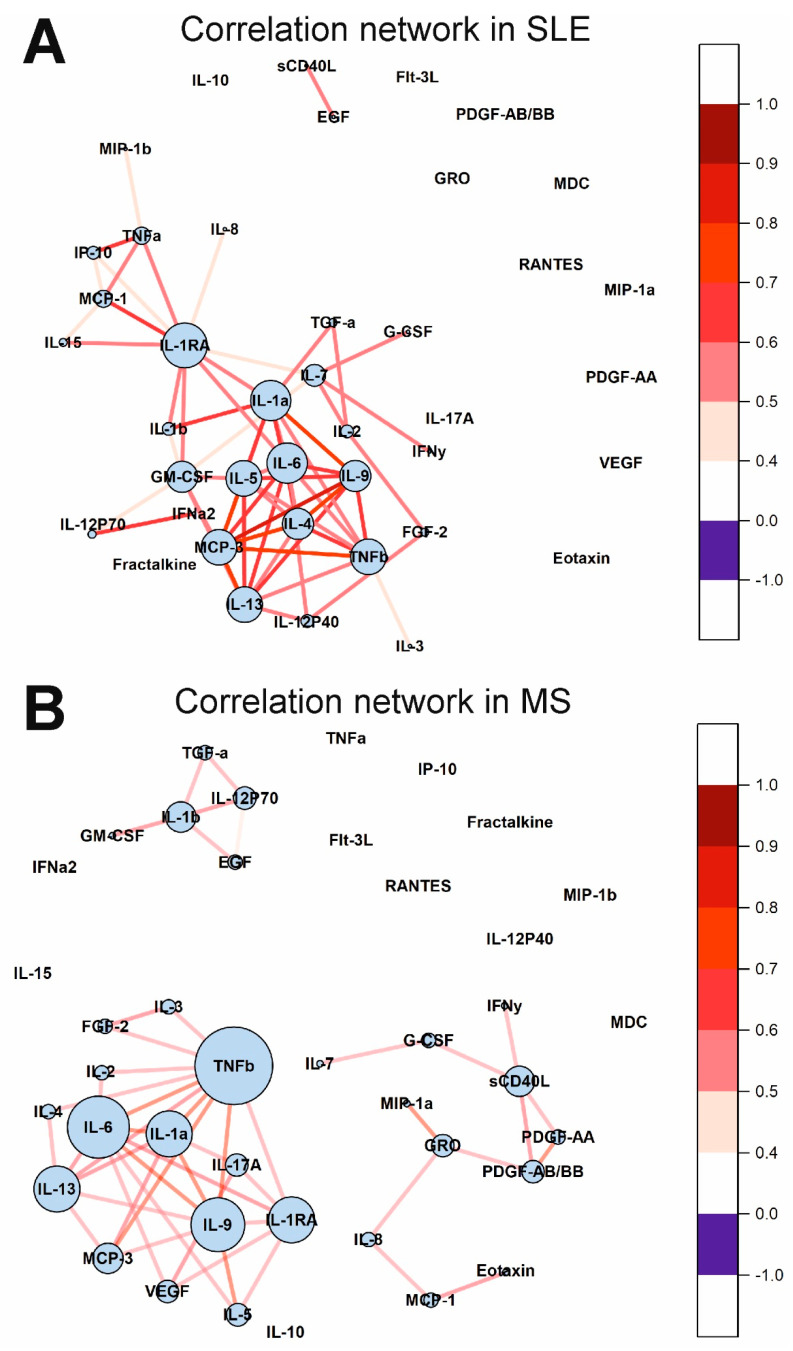
Cytokine correlation networks in SLE (**A**) and MS (**B**). Correlations between concentrations of different cytokines are shown as colored lines. A scale showing the strength and direction of the correlation is shown in the right panel. Each cytokine is represented by a blue circle whose size is proportional to the number of statistically significant correlations.

The cytokine correlation networks in the SLE and MS groups are shown in Figure 7. Many cytokines correlate in SLE and MS. Interestingly, all correlations are positive. It is important to note that large correlation clusters are observed in SLE and MS. There are three large clusters in MS. Cytokines such as IL-1RA, IL-1a, IL-6, TNFb, and others, in SLE and TNFb, IL-6, IL-9, IL-1RA, IL-13, and others, in MS, have many correlations and form a correlation network. Thus, the cytokine correlation networks differ in SLE, MS, and healthy individuals.

Correlation analysis of cytokine levels with clinical data revealed only some significant correlations (see Supplementary Tables S5–S7). In the HS group, GRO and IP-10 levels were positively correlated with age (r = 0.473, *p* = 0.003 and r = 0.642, *p* = 0.00002, respectively) (Supplementary Table S5). In the SLE group, MDC concentration was positively correlated with body mass index (BMI) (r = 0.445, *p* = 0.005), and IL-5 level was negatively correlated with high-density lipoprotein (HDL) concentration (r = 0.461, *p* = 0.003) (Supplementary Table S6). Importantly, IFNa2 concentration was positively correlated (r = 0.456, *p* = 0.005) with the SELENA-SLEDAI index score (Supplementary Table S6). IL-10 and MCP-1 levels were positively correlated with the SELENA-SLEDAI index score, but to a lesser extent (r = 0.358, *p* = 0.01 and r = 0.349, *p* = 0.011, respectively). In the MS group, no significant correlations with the EDSS index score were found (Supplementary Table S7). The correlation of GRO and PDGF-AA/BB levels with age (r = 0.433, *p* = 0.002 and r = 0.396, *p* = 0.006, respectively) is the only one that has been revealed.

## 3. Discussion

### 3.1. Cytokine Profiles in SLE

The results obtained revealed that cytokine profiles are significantly different in SLE and MS when compared with healthy people and between diseases (see Table 2, Figure 1, Figure 2, Figure 3 and Figure 5). However, it is important to note that this study included SLE patients receiving therapy. Pathogenetic therapy for SLE involves a direct effect on the human immune system, so such intervention may affect the cytokine profiles of patients [37,38]. Despite this, the absolute serum concentrations of 11 cytokines in patients with SLE were higher than in healthy donors (see Figure 1 and Table 2). All these cytokines can be divided into interleukins, TNF superfamily members, and chemokines. Patients with SLE show a significant increase in classical pro- and anti-inflammatory cytokines involved in the pathogenesis of SLE, including TNF-α (*p* = 1.3 × 10^−4^), IL-1b (*p* = 0.005), IL-6 (*p* = 8.2 × 10^−5^), and IL-10 (*p* = 0.005). The increase in these cytokines in SLE is consistent with the literature data [39,40,41]. The increase in IL-1RA (*p* < 0.0001) may be a response to the increase in IL-1b. IL-10 and IL-9 have anti-inflammatory functions [42], so their increase may be a compensatory response to an increase in pro-inflammatory cytokines. There is evidence that IL-15 is elevated in SLE regardless of disease activity [43]. Therefore, the obtained data on the increase in the level of IL-15 in SLE is consistent with the literature data. Our findings of decreased sCD40L levels were unexpected, as SLE is usually associated with elevated sCD40L levels [44]. This result can be explained by the effect of therapy. In addition, several chemokines including MCP-1/CCL2 (*p* = 0.007), MIP-1a/CCL3 (*p* = 0.007), MIP-1b/CCL4 (*p* = 0.001), and fractalkine/CX3CL1 (*p* = 0.0001), were increased in SLE. At the same time, the level of MDC/CCL22 decreased (*p* = 0.002). These cytokines are involved in the process of recruiting immunocompetent cells such as monocytes, macrophages, natural killers, dendritic cells, and T-lymphocytes, which, in turn, are involved in the SLE pathogenesis [45,46]. Many of these cells themselves secrete these cytokines, which leads to the formation of a pathogenic loop. The secretion of MIP-1a/CCL3 and MIP-1b/CCL4 is also stimulated by the pro-inflammatory cytokine IL-1b, the concentration of which is also significantly higher in SLE patients (*p* = 0.005) than in healthy donors. Multiple observations of increased levels of MCP-1/CCL2, MIP-1a/CCL3, MIP-1b/CCL4, and fractalkine/CX3CL1 in SLE support our findings [47,48,49]. Thus, these data indicate that not only changes in pro- and anti-inflammatory cytokines, but also chemokines play an essential role in the pathogenesis of SLE.

Interestingly, the levels of several cytokines have been associated with the clinical parameters of SLE. IL-10 and MCP-1/CCL2 levels were higher in patients with a SELENA-SLEDAI index score of more than 7, suggesting that levels of these cytokines increase with increasing severity of SLE. This is also confirmed by the results of the correlation analysis (Supplementary Table S6). IL-10 and MCP-1 levels were positively correlated with the SELENA-SLEDAI index score (r = 0.358, *p* = 0.01 and r = 0.349, *p* = 0.011, respectively). SLE is known to be characterized by the activation of the interferon pathway [50]. Therefore, our data on the positive correlation of the IFNa2 level with the SELENA-SLEDAI index score confirm the important role of the interferon pathway in SLE. In addition, we found associations between cytokine levels and disease duration. In particular, the levels of IFNy, IL-10, IL-17A, and IL-2 decreased, and the level of IL-8/CXCL8 increased with the course of the disease. A decrease in cytokines may be associated with the influence of therapy, and an increase in IL-8/CXCL8 may indicate the involvement of the central nervous system in long-term SLE [47].

The results of protein interaction network analysis showed that all significantly altered cytokines in SLE are functionally interconnected (Figure 4A). Figure 4A clearly shows the functional associations in the disrupted cytokine network in SLE. Thus, dysregulation of the cytokine network in treated SLE patients is primarily associated with changes in the functional relationships of TNFa, IL-1b, IL-6, IL-10, and MCP-1/CCL2. Alterations in these core cytokines contribute to the dysregulation of other cytokines and chemokines including MIP-1a/CCL3, MIP-1b/CCL4, fractalkine/CX3CL1, sCD40L, MDC/CCL22, IL-15, PDGF-A, and IL-1RA. Taken together, these data indicate that cytokine dysregulation in SLE involves interleukins, TNF superfamily members, and chemokines, which are functionally interrelated.

Investigation of changes in correlations between cytokines in various diseases can provide valuable information about regulatory interactions in cytokine networks. We showed that the number of statistically significant correlations between cytokines increased by 5.5 times in SLE than in healthy donors (see Figure 6 and Figure 7). In the case of healthy donors, only individual cytokines correlate with each other and do not form large clusters (Figure 6). By contrast, in the case of SLE, almost all statistically significant correlations form one supercluster of correlation relationships (Figure 7A). All this visually represents and confirms the systemic nature of the processes occurring in SLE. The key cytokines holding the cluster together are the cytokines from the IL-1 family, namely IL-1RA, IL-1a, and, to a lesser extent, IL-1b. In addition, IL-6, IL-9, and IL-13 play an important role in intercytokine interaction. A number of studies have demonstrated an association between the level of IL-1 group cytokines and SLE activity [51,52,53,54]. Thus, the correlation analysis demonstrated a change in the regulatory interactions between cytokines in SLE.

### 3.2. Cytokine Profiles in MS

Analysis of cytokine profiles in untreated MS patients showed that although some cytokines (TNFb, IL-1a, and IL-9) were increased in MS, these changes were not significant after Benjamini–Hochberg correction. Therefore, all significantly changed cytokines in MS patients were significantly lower than in healthy donors (see Figure 2 and Table 2). Unlike SLE, which is characterized by high levels of some cytokines, even during treatment (see Section 4.1), the concentration of many cytokines in MS does not differ from the control or decreases. The observed trend may be related to the low level of disability in patients included in the study (median EDSS Index Score was 2.5). All decreased cytokines in MS patients can be divided into two large groups: growth factors, VEGF (*p* = 3.6 × 10^−4^), PDGF-AB/BB (*p* = 1.1 × 10^−6^), TGF-a (*p* = 1.6 × 10^−7^), EGF (*p* = 7.7 × 10^−18^), and PDGF-AA (*p* = 2.6 × 10^−15^), and chemokines, IL-8/CXCL8 (*p* = 7.1 × 10^−6^), MDC/CCL22 (*p* = 5.5 × 10^−3^), MIP-1b/CCL4 (*p* = 1.5 × 10^−3^), and MCP-1/CCL2 (*p* = 4.1 × 10^−3^). The observed decrease in growth factors, such as EGF, PDGF, and VEGF, in MS is consistent with the literature data [55,56]. Scalabrino et al. showed that EGF levels were reduced and did not depend on the course of MS [26]. PDGF and EGF levels negatively correlated with the survival of patients with MS [55,57,58]. EGF and PDGF also stimulate remyelination of chronic lesions [26,59], so their decreased level may reduce the activity of reparative processes in patients with MS. IFNα2 may also play a protective role in MS [60]. We found that IFNα2 and PDGF-AB/BB levels were significantly higher in PPMS than in RRMS. This may be a compensatory response to more severe PPMS than RRMS. There is also evidence that the level of TGF-a and the TGF-a/VEGF ratio was reduced in RRMS and correlated with neurological impairment [61]. Our data also confirm these observations (the median TGF-a/VEGF ratio was 0.032 in the HS group and 0.029 in the MS group). TGF-a and VEGF imbalances affect astrocytes and stimulate neuroinflammation [61]. TGF-a and VEGF expression are regulated by the aryl hydrocarbon receptor, which binds polycyclic and aromatic hydrocarbons and thereby mediates the influence of environmental and microbial factors [61]. Thus, the decrease in TGF-a and TGF-a/VEGF ratio in MS may reflect the influence of environmental factors.

We have found abnormalities in the levels of not only growth factors but also chemokines in MS. MCP-1/CCL2 is a potent chemokine for macrophage recruitment. Serum CCL2 levels were significantly lower in patients with acute RRMS with Gd-enhanced lesions on MRI than in patients without enhanced lesions [62]. The decrease in CCL2 that we found may be an indicator of an increase in disease activity. Increased CCL4 may induce hyperpermeability of the blood-brain barrier [63]. However, we found a decrease in CCL4 levels, which is consistent with another observation in MS [64]. MDC/CCL22 is a chemokine that attracts CCR4-expressing cells including Th2/Treg cells to an inflammatory site. Th2 cells produce anti-inflammatory cytokines, so CCL22 plays a protective role in MS [24]. There is evidence of a decrease in serum CCL22 levels in women with MS [65]. We also found a decrease in CCL22 concentration, but the differences between men and women were not significant.

Protein interaction network analysis also confirmed the important role of growth factors and chemokines in MS (Figure 4B). It can be assumed that the impaired functional relationship between growth factors and chemokines is an essential feature of the cytokine network in MS.

Cytokine sCD40L is not a growth factor or chemokine, but its level also decreased (*p* = 1.6 × 10^−21^) in patients with MS. The main function of sCD40L is associated with the stimulation of B cell proliferation and blocking B cell apoptosis [66]. A decrease in sCD40L may be associated with impaired non-hemostatic functions of platelets in MS since a decrease in sCD40L correlates with a decrease in PDGF-AA and PDGF-AB/BB, which are mainly produced by platelets [67,68].

IFNg, IL-17, GM-CSF, and TNFα are believed to be actively involved in the pathogenesis of MS [23]. However, in our study, we did not find significant changes in these cytokines.

Correlation analysis showed that the number of correlations between cytokines increases by 4.5 times for MS compared to healthy donors (Figure 6 and Figure 7). Unlike SLE, where the number of connections between large nodes of the correlation network is distributed evenly, the largest connecting element in MS is TNFβ, which has connections with many cytokines in the cluster (Figure 7B). There is evidence that TNF superfamily proteins are associated with inflammation and demyelination in MS [69]. Therefore, TNF superfamily proteins may play an important role in altering the regulatory interactions between cytokines in MS.

### 3.3. Cross-Disorder Analysis

Cross-disorder analysis of cytokine levels in SLE and MS revealed changes in 22 cytokines (see Figure 3 and Table 2). Levels of all 22 cytokines decreased in MS. Most of these cytokines were also reduced in MS compared with healthy donors. But eotaxin, G-CSF, GRO/CXCL1, IFNγ, IL-7, and IL-17A, were elevated in SLE compared with MS (Table 2). An increase in these cytokines may distinguish the pathological process in SLE from that in MS. Future research may focus on the role of these cytokines in SLE and MS. However, the treatment of patients with SLE may have influenced these results, so they should be tested in future studies.

### 3.4. Limitations

This work has limitations, just as with any study. Therefore, the results obtained must be interpreted with caution. First, the sample of healthy donors and patients was relatively small. Therefore, we could miss significant differences for some cytokines (type II error). However, we still found a number of statistically significant differences. Second, the treatment of SLE patients could affect the levels of many cytokines. Therefore, further studies are needed to find out what caused the detected changes. Third, the group of SLE patients was not comparable with other groups in terms of sex. Women are known to have a higher prevalence of SLE, so more women were included in the study. It was very difficult to recruit the same number of men with SLE. However, we did not find differences in cytokine levels between men and women with SLE.

## 4. Materials and Methods

### 4.1. Design of the Study

This work is a case-control and cross-disorder study. We compared the cytokine profile in groups of SLE and MS patients with a group of healthy donors, as well as groups of SLE and MS patients. The study was carried out in accordance with the Ethical Principles for Medical Research Involving Human Subjects (World Medical Association Declaration of Helsinki, 1964–2013) [70] and approved by the Ethics Committee of the Institute of Chemical Biology and Fundamental Medicine (protocol N8 from 7 February 2020). All participants provided written informed consent.

### 4.2. Patients and Healthy Subjects

The healthy donors and patients were recruited at the Institute of Clinical Immunology (Novosibirsk, Russia) between February 2020 and March 2022. Sixty patients with SLE were selected for the SLE group. Inclusion criteria for SLE patients were as follows: signed consent, over 18 years of age, and formally diagnosed with SLE. The diagnosis of SLE (M32, ICD-10) was made in accordance with the Russian Association of Rheumatologists and EULAR recommendations [71] based on laboratory and physical tests (as in [72]). The SELENA-SLEDAI scale was applied to evaluate SLE disease activity [73].

Fifty-eight patients were included in the MS group. Inclusion criteria for MS patients were as follows: signed consent, over 18 years of age, and formally diagnosed with MS. The diagnosis of MS (G35, ICD-10) was made in accordance with national and international (McDonald Criteria 2017) recommendations [74]. The Expanded Disability Status Scale (EDSS) was used to evaluate disability progression in MS [75].

It is important to note that patients with SLE received therapy that affects the immune system. Every SLE patient received at least two drugs, one of which was a steroid (prednisolone, methylprednisolone, betamethasone, or dexamethasone). Other drugs that affect the immune system included hydroxychloroquine, methotrexate, mycophenolate mofetil, azathioprine, filgrastim, celecoxib, and tenoxicam. Dosage information for each drug is given in Supplementary Table S1. Patients with MS did not receive any therapy. The blood sampling of MS patients for the study was carried out immediately after admission to the hospital, before the start of specific treatment.

In addition, thirty-six people were selected for the HS group. The inclusion criteria for the HS group were as follows: signed consent and over 18 years of age. Other information is shown in Table 1.

The exclusion criteria for SLE and MS patients and healthy individuals were as follows: lupus nephritis (for SLE), diabetes, obesity, metabolic syndrome, drug addiction, the presence of concomitant autoimmune disease, oncological disease, or other concomitant somatic diseases in the acute stage. Vaccinations, infectious diseases, or allergic reactions in the previous month, were also exclusion criteria.

### 4.3. Collection of Serum Samples

Blood samples were collected from each subject after eight hours of overnight fasting by venipuncture in a BD Vacutainer PET tube with a clot activator (silica) (BD, Franklin Lakes, NJ, USA). The collected blood samples were centrifuged at 2000× *g* at 4 °C for 30 min. The serum was aliquoted into plastic tubes and stored in an ultra-low temperature freezer at −80 °C until analysis. Multiple freeze-thaw cycles were avoided.

### 4.4. Multiplex Serum Cytokine Immunoassay

All measurements were carried out at the Core Facility “Medical genomics” of the Tomsk National Research Medical Center. The serum cytokine concentration was determined by a MAGPIX^®^ Multiplexing Instrument (Luminex, Austin, TX, USA) using the MILLIPLEX MAP Human Cytokine/Chemokine Magnetic Bead Panel-Premixed 41 Plex, Cat. N. HCYTMAG-60K-PX41 (Merck Millipore, Darmstadt, Germany). A list of abbreviations used, and descriptions of the forty-one analyzed cytokines, are provided in Appendix Table A1. All measurement steps were carried out in accordance with the manufacturer’s recommendations. The analysis of the detected signals was carried out in Luminex xPONENT^®^ software. Further analysis is performed in MILLIPLEX^®^ Analyst 5.1 software. The concentration of cytokines was presented as pg/mL.

### 4.5. Statistical Analysis

Statistical data processing was carried out in OriginPro 2021 (OriginLab, Northampton, MA, USA) and STATISTICA 10 (StatSoft, Tulsa, OK, USA). The Shapiro–Wilk test was used to assess the normality of the data distribution. Most of the variables had a non-normal distribution, so non-parametric tests were used. Pearson’s chi-squared test was used to analyze the categorical variables. The significance of the differences among multiple groups was calculated using the Kruskal–Wallis test followed by Dunn’s post hoc test for multiple comparisons. The Benjamini–Hochberg procedure was used for multiple comparisons of correction. Differences were considered significant only for variables that remained significant after Benjamini–Hochberg correction. Partial Least-Squares Discriminant Analysis (PLS-DA) was carried out using the online tool CytokineExplore (http://al-saleh.cc/exabx.com/apps/cytokineexplore/, accessed on 20 August 2022) [35]. The correlation analysis was carried out using Spearman’s rank correlation test with Benjamini–Hochberg multiple comparisons correction. Correlation coefficients with a *p*-value remaining significant after Benjamini–Hochberg correction were considered statistically significant. OriginPro 2021 (OriginLab, Northampton, MA, USA) и GraphPad Prism 7 (GraphPad Software, San Diego, CA, USA) was used for plotting.

### 4.6. Protein-Protein Interaction Network Analysis

The STRING 11 online tool (https://version-11-0.string-db.org/ (accessed on 29 September 2022) [34] was used to construct protein-protein interaction networks of the significantly altered cytokines between different groups (as in [64]). The graph was based on data on protein-protein associations including activation, inhibition, binding, transcriptional regulation, catalysis, posttranslational modification, etc. The medium interaction score (0.4) was used for STRING analysis. The network is clustered using k-means with a k of 3.

## 5. Conclusions

In this work, we identified characteristic differences in the cytokine profiles of treated patients with MS and SLE compared with healthy donors and between diseases. Changes in the cytokine profile in SLE have been associated with the dysregulation of interleukins, TNF superfamily members, and chemokines. Dysregulation of the cytokine network in MS is characterized by impaired functional relationships between growth factors and chemokines. In addition, all altered cytokines in SLE and MS are functionally interrelated. Therefore, therapeutic strategies aimed at suppressing only one cytokine may be ineffective or have side effects. New therapeutic agents should be aimed at normalizing the entire cytokine network in SLE and MS.

## Figures and Tables

**Table 1:** Clinical characteristics of the studied groups of patients and healthy individuals.

Parameters	HS Group	SLE Group	MS Group	Statistics *
Number of participants	36	60	56	–
Age, years Me [Q1–Q3], (Min-Max)	36.5 [28–58.25] (24–65)	38.5 [28.8–59] (21–72)	37 [26.8–43.3] (20–57)	N.S.
Sex, F/M, %	50/50	83.3/16.7	52.7/47.3	HS vs. SLE: *p* < 0.0001 HS vs. MS: N.S. SLE vs. MS: *p* < 0.0001
Disease duration, years Me [Q1–Q3], (Min–Max)	–	7 [4–16.3] (1–25)	3 [1–7.8] (1–23)	SLE vs. MS: *p* = 0.0002
SELENA-SLEDAI Index Score Me [Q1–Q3], (Min–Max)	–	6.5 [4,5,6,7,8,9,10] (1–18)	–	–
Exacerbation: Yes/No, n	–	51/9	–	–
EDSS Index Score Me [Q1–Q3], (Min–Max)	–	–	2.5 [1.5–3.5] (1–5.5)	–
MS type: PPMS/RRMS, n	–	–	20/36	–
MS phase: Stable/aggravation/ remission/N.D., n	–	–	14 (PPMS)/16 (RRMS)/20 (RRMS)/6 (PPMS)	–
Patients received therapy, %	–	100%	0	–

* The significance of the differences was calculated using the Kruskal–Wallis ANOVA test or Pearson’s chi-squared test (for sex). A *p*-value > 0.05 was considered not significant (N.S.). Abbreviations: N.D.–No Data, SELENA-SLEDAI–Safety of Estrogens in Lupus Erythematosus: National Assessment–Systemic Lupus Erythematosus Disease Activity Index, EDSS–Expanded Disability Status Scale, PPMS–Primary Progressive Multiple Sclerosis, RRMS–Relapsing-remitting Multiple Sclerosis.

**Table 2:** Summary of cytokines whose serum levels changed significantly in the analyzed groups.

HS vs. SLE			HS vs. MS			SLE vs. MS		
Cytokine *	*p*-Value	Comment	Cytokine *	*p*-Value	Comment	Cytokine *	*p*-Value	Comment
sCD40L	0.002	Decreased in SLE	sCD40L	<0.0001	Decreased in MS	sCD40L	<0.0001	Decreased in MS
MCP-1/CCL2	0.007	Increased in SLE	MCP-1/CCL2	0.004	Decreased in MS	MCP-1/CCL2	<0.0001	Decreased in MS
MIP-1b/CCL4	0.01	Increased in SLE	MIP-1b/CCL4	0.001	Decreased in MS	MIP-1b/CCL4	<0.0001	Decreased in MS
PDGF-AA	0.006	Decreased in SLE	PDGF-AA	<0.0001	Decreased in MS	PDGF-AA	<0.0001	Decreased in MS
MDC/CCL22	0.002	Decreased in SLE	MDC/CCL22	0.005	Decreased in MS	IL-1b	0.001	Decreased in MS
IL-1b	0.005	Increased in SLE	EGF	<0.0001	Decreased in MS	IL-1RA	<0.0001	Decreased in MS
IL-1RA	<0.0001	Increased in SLE	IL-8/CXCL8	<0.0001	Decreased in MS	IL-6	0.0008	Decreased in MS
IL-6	<0.0001	Increased in SLE	TGF-a	<0.0001	Decreased in MS	IL-15	<0.0001	Decreased in MS
IL-15	<0.0001	Increased in SLE	PDGF-AB/BB	<0.0001	Decreased in MS	EGF	<0.0001	Decreased in MS
MIP-1b/CCL3	0.007	Increased in SLE	VEGF	0.0003	Decreased in MS	MIP-1b/CCL4	<0.0001	Decreased in MS
IL-10	0.005	Increased in SLE	–	–	–	IL-10	0.01	Decreased in MS
TNFa	<0.001	Increased in SLE	–	–	–	TNFa	<0.0001	Decreased in MS
Fractalkine/CX3CL1	<0.001	Increased in SLE	–	–	–	IL-8/CXCL8	<0.0001	Decreased in MS
IL-9	0.001	Increased in SLE	–	–	–	PDGF-AB/BB	<0.0001	Decreased in MS
–	–	–	–	–	–	TGF-a	<0.0001	Decreased in MS
–	–	–	–	–	–	VEGF	<0.0001	Decreased in MS
–	–	–	–	–	–	Eotaxin/CCL11	0.0001	Decreased in MS
–	–	–	–	–	–	G-CSF	0.006	Decreased in MS
–	–	–	–	–	–	GRO/CXCL1	0.0003	Decreased in MS
–	–	–	–	–	–	IFNy	0.0001	Decreased in MS
–	–	–	–	–	–	IL-7	<0.0001	Decreased in MS
–	–	–	–	–	–	IL-17A	0.004	Decreased in MS

* The same cytokines that significantly change in different comparison sets are shown in the same color.

## Data Availability

The datasets analyzed during the current study are available from the corresponding author on reasonable request. The copyright for the datasets obtained in this work is registered with the Federal Service for Intellectual Property of Ministry of Economic Development of the Russian Federation (certificate N 2022620287; the copyright holder is the Institute of Chemical Biology and Fundamental Medicine). Reuse of datasets is possible with the permission of the copyright holder. The published data can be used without restrictions.

## Supplementary Materials

The supporting information can be downloaded at: https://www.mdpi.com/article/10.3390/ijms232213829/s1.

## Appendix A

**Table A1:** Description of cytokines analyzed in the study.

Abbreviation	Description	Family
EGF	Epidermal growth factor	Growth factors
Eotaxin/CCL11	C-C motif chemokine ligand 11, eosinophil chemotactic protein	Chemokines
FGF2	Fibroblast growth factor 2 or Basic fibroblast growth factor	Growth factors
Flt-3L	Fms-related tyrosine kinase 3 ligand	Growth factors
Fractalkine/CX3CL1	Fractalkine, C-X3-C motif chemokine ligand 1	Chemokines
G-CSF	Granulocyte colony-stimulating factor	Growth factors
GM-CSF	Granulocyte-macrophage colony-stimulating factor	Growth factors
GRO/CXCL1	Growth-regulated alpha protein, C-X-C motif chemokine ligand 1	Chemokines
IFNa2	Interferon α2	Interferons
IFNy	Interferon γ	Interferons
IL-1a	Interleukin-1α	Interleukins
IL-1b	Interleukin-1β	Interleukins
IL-1RA	Interleukin-1 receptor antagonist	Interleukins
IL-2	Interleukin-2	Interleukins
IL-3	Interleukin-3	Interleukins
IL-4	Interleukin-4	Interleukins
IL-5	Interleukin-5	Interleukins
IL-6	Interleukin-6	Interleukins
IL-7	Interleukin-7	Interleukins
IL-8/CXCL8	Interleukin-8, C-X-C motif chemokine ligand 8	Interleukins/Chemokines
IL-9	Interleukin-9	Interleukins
IL-10	Interleukin-10	Interleukins
IL-12P40	Subunit beta of interleukin 12, natural killer cell stimulatory factor 2, cytotoxic lymphocyte maturation factor p40, interleukin-12 subunit p40	Interleukins
IL-12P70	Interleukin-12 consisting of two covalently linked subunits p40 and p35	Interleukins
IL-13	Interleukin-13	Interleukins
IL-15	Interleukin-15	Interleukins
IL-17A	Interleukin-17A	Interleukins
IP-10/CXCL10	Interferon gamma-induced protein 10, C-X-C motif chemokine ligand 10	Chemokines
MCP-1/CCL2	Monocyte chemoattractant protein 1, C-C motif chemokine ligand 2	Chemokines
MCP-3/CCL7	Monocyte-chemotactic protein 3, C-C motif chemokine 7	Chemokines
MDC/CCL22	C-C motif chemokine 22, macrophage-derived chemokine	Chemokines
MIP-1a/CCL3	Macrophage inflammatory protein-1α, C-C motif chemokine ligand 3	Chemokines
MIP-1b/CCL4	Macrophage inflammatory protein-1β, C-C motif chemokine ligand 4	Chemokines
PDGF-AA	Platelet-derived growth factor AA	Growth factors
PDGF-AB/BB	Platelet-derived growth factor AB/BB	Growth factors
RANTES/CCL5	Regulated on activation, normal T cell expressed and secreted (RANTES); C-C motif chemokine ligand 5	Chemokines
sCD40L	Soluble CD-40 ligand, tumor necrosis factor ligand superfamily member 5	TNF superfamily members
TGF-a	Transforming growth factor α	Growth factors
TNFα	Tumor necrosis factor α	TNF superfamily members
TNFb	Tumor necrosis factor β	TNF superfamily members
VEGF	Vascular endothelial growth factor	Growth factors
