# Supplementary material for: Multiplex Analysis of Serum Cytokine Profiles in Systemic Lupus Erythematosus and Multiple Sclerosis
Source: Int J Mol Sci. 2022 Nov 10;23(22):13829. doi: 10.3390/ijms232213829 (PMC9695219; doi:10.3390/ijms232213829)
Supplement: Supplementary file 1 [file ijms-23-13829-s001.zip › Supplement Figures.pdf]

Supplement Figures

Article

Multiplex Analysis of Serum Cytokine Profiles in Systemic Lupus Erythematosus and Multiple Sclerosis

Mark M. Melamud [1,†], Evgeny A. Ermakov [1,†], Anastasiia S. Boiko [2], Daria A. Kamaeva [2], Alexey E. Sizikov [3], Svetlana A. Ivanova [2], Natalia M. Baulina [4], Olga O. Favorova [4], Georgy A. Nevinsky [1] and Valentina N. Buneva 1,*

[1] Institute of Chemical Biology and Fundamental Medicine, Siberian Branch of the Russian Academy of Sciences, 630090 Novosibirsk, Russia
[2] Mental Health Research Institute, Tomsk National Research Medical Center of the Russian Academy of Sciences, 634014 Tomsk, Russia
[3] Institute of Clinical Immunology, Siberian Branch of the Russian Academy of Sciences, 630099 Novosibirsk, Russia
[4] Pirogov Russian National Research Medical University, 117997 Moscow, Russia
* Correspondence: buneva@niboch.nsc.ru (V.N.B.); Tel.: +7-383-363-51-27
† These authors contributed equally to this work.

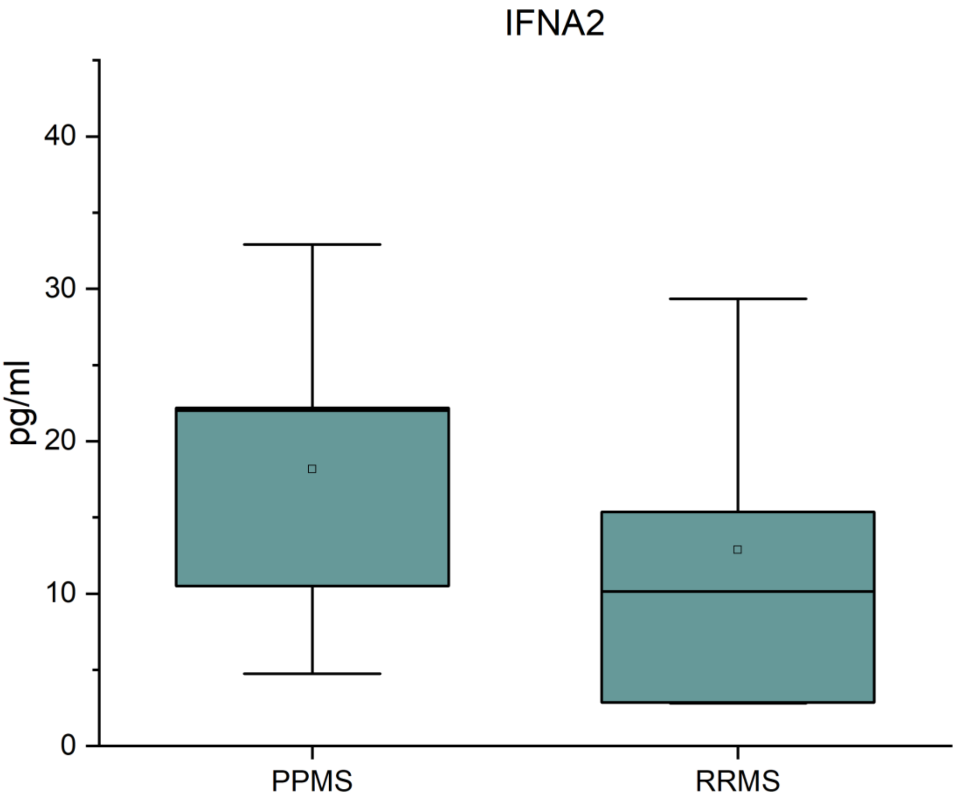

Figure S1. The concentration of IFNα2 in the serum of patients depending on the type of multiple sclerosis (n = 20 for PPMS and n = 36 for RRMS). PPMS – Primary Progressive Multiple Sclerosis, RRMS – Relapsing-remitting Multiple Sclerosis.

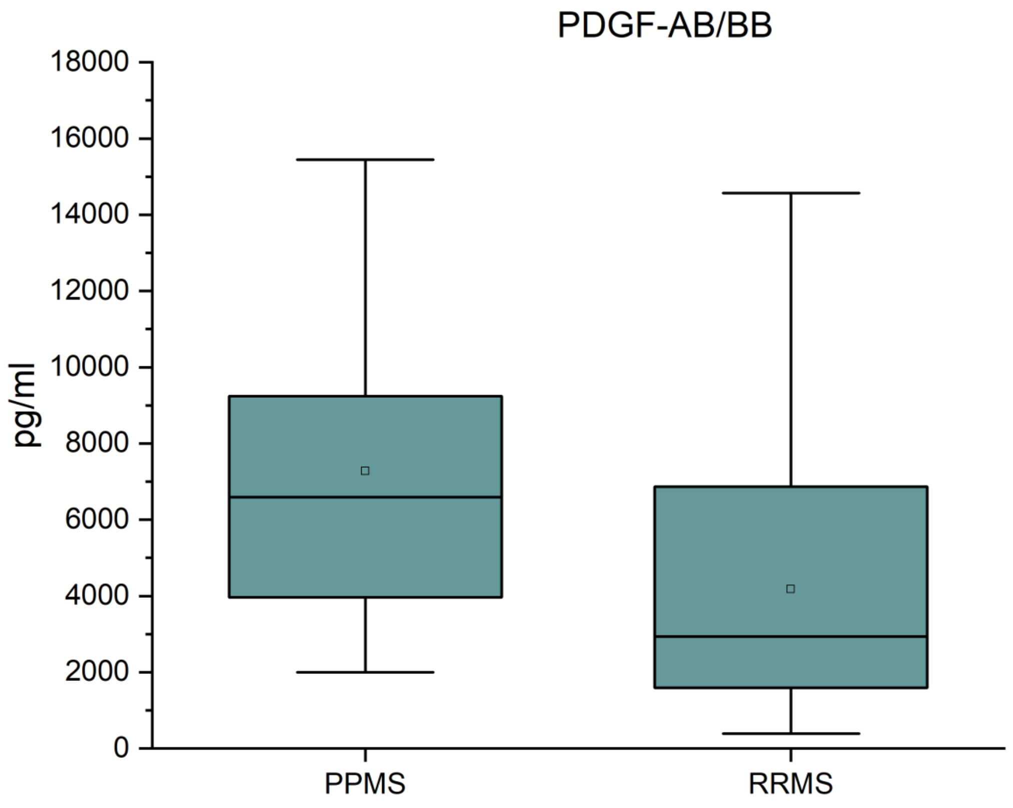

Figure S2. The concentration of PDGF-AB/BB in the serum of patients depending on the type of multiple sclerosis (n = 20 for PPMS and n = 36 for RRMS). PPMS – Primary Progressive Multiple Sclerosis, RRMS – Relapsing-remitting Multiple Sclerosis.

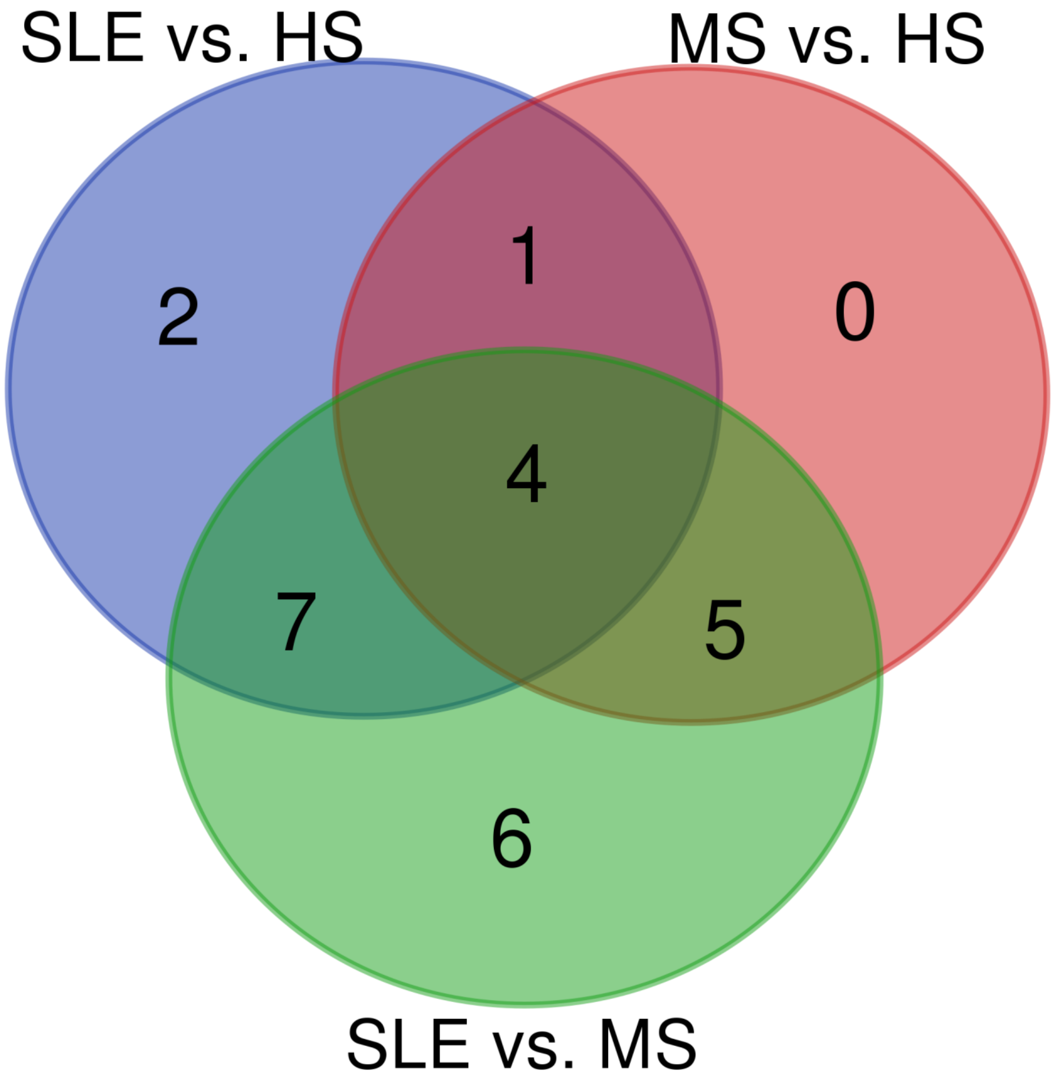

Figure S3. Venn diagram showing the number of overlapping significantly changed cytokines in different comparison groups.

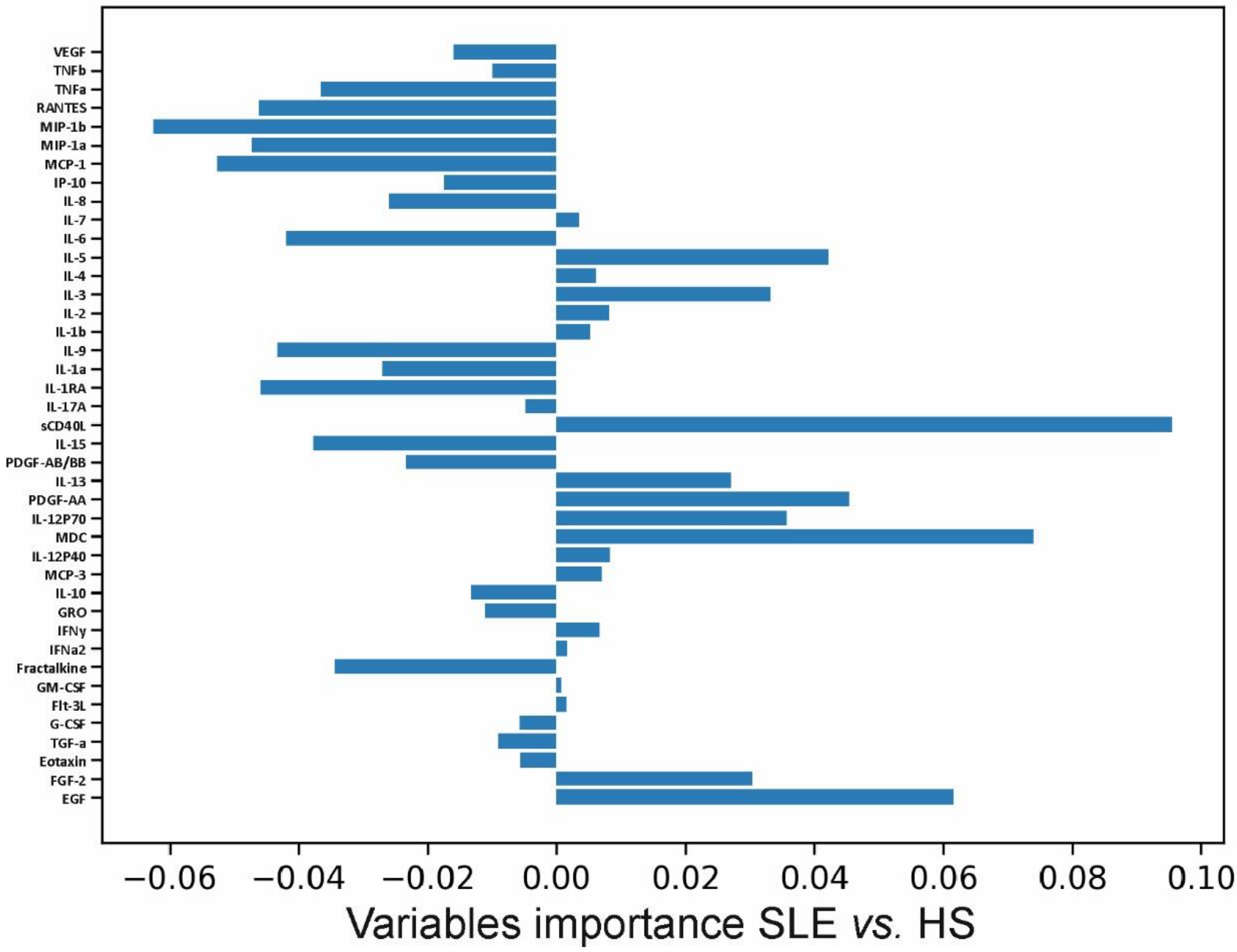

Figure S4. Variables importance in PLS-DA comparing SLE vs. HS groups.

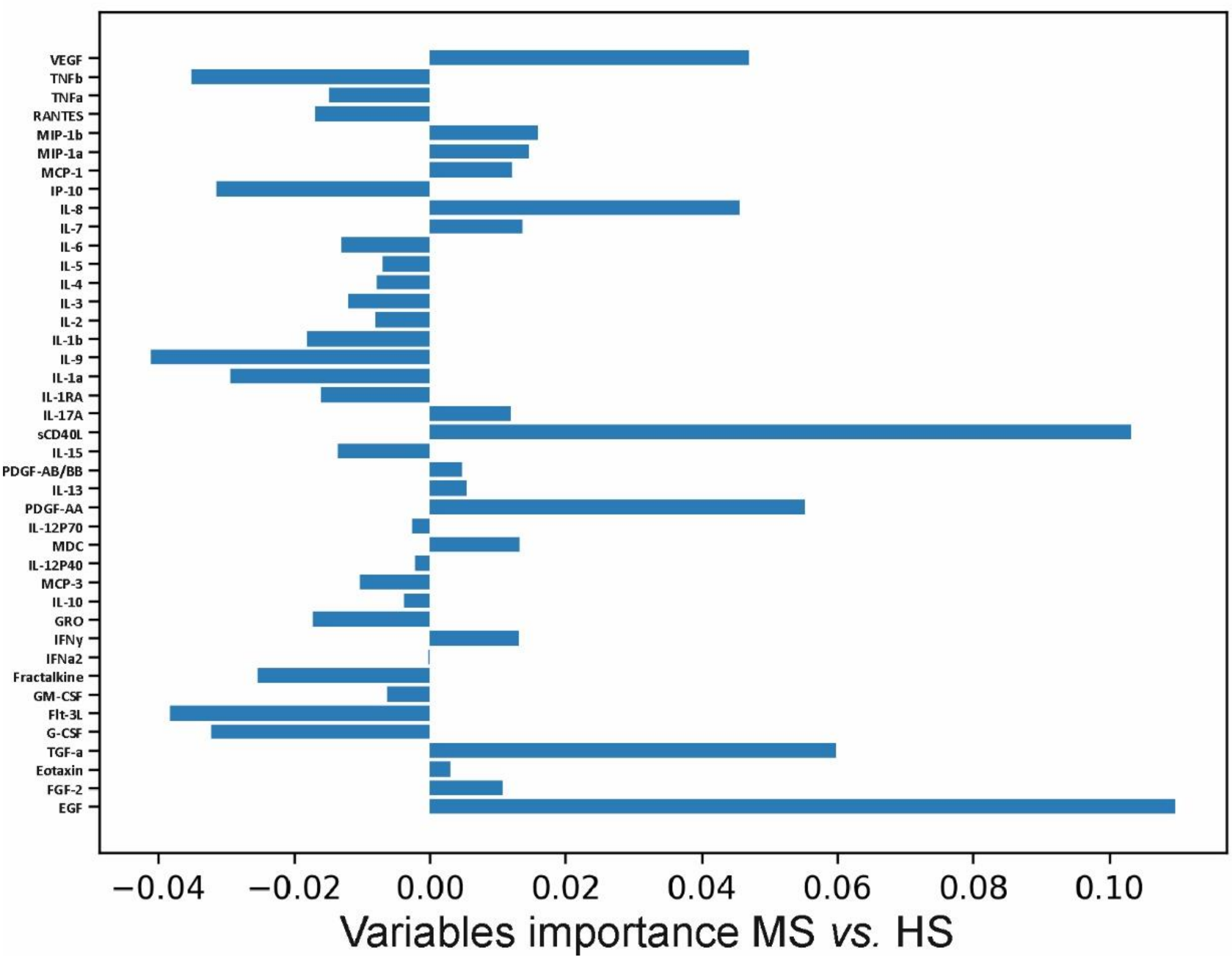

Figure S5. Variables importance in PLS-DA comparing MS vs. HS groups.

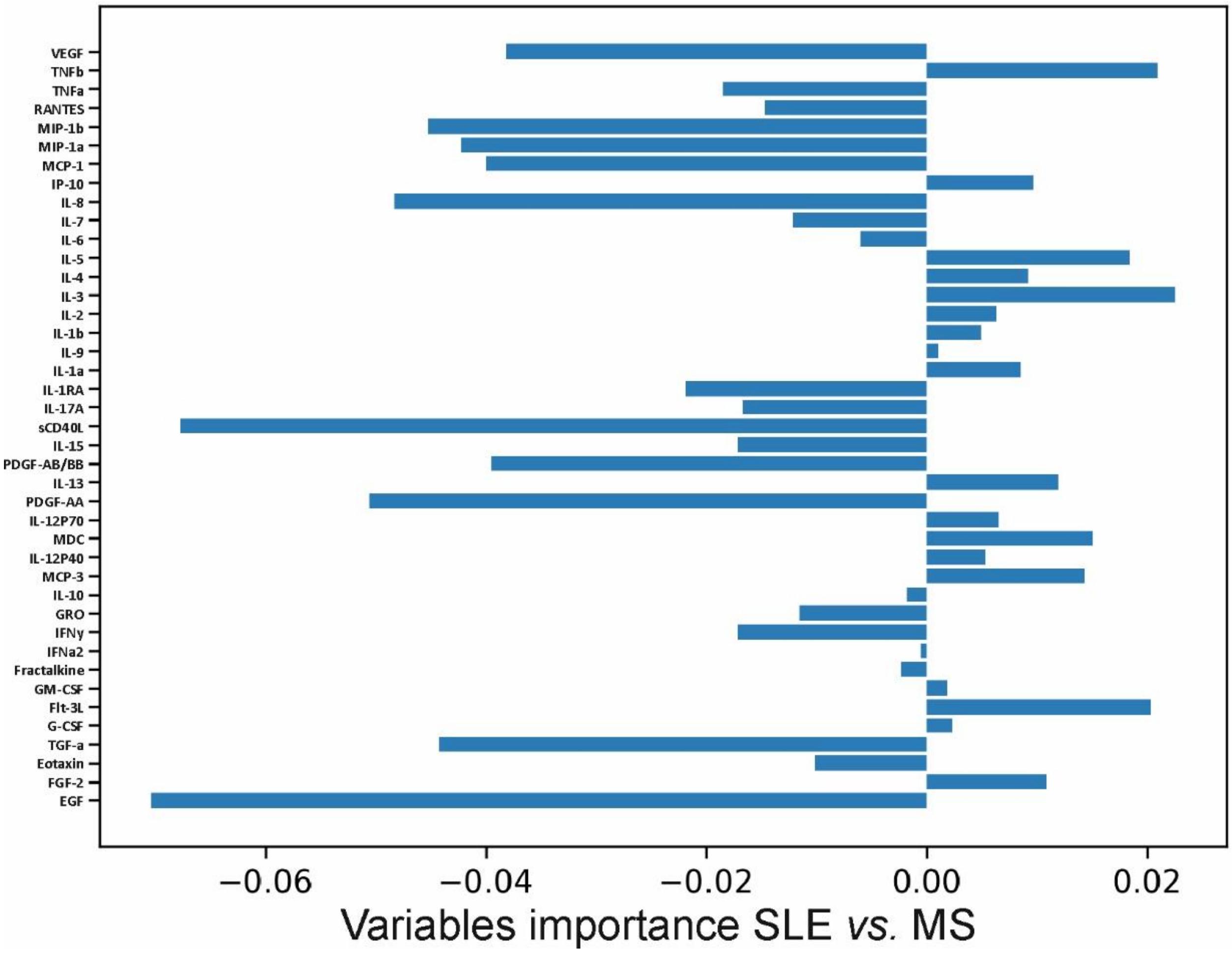

Figure S6. Variables importance in PLS-DA comparing SLE vs. MS groups.
